# Supplementary material for: Buccal Bone Thickness in Anterior and Posterior Teeth—A Systematic Review
Source: Healthcare (Basel). 2021 Nov 30;9(12):1663. doi: 10.3390/healthcare9121663 (PMC8700878; doi:10.3390/healthcare9121663)
Supplement: Supplementary file 1 [file healthcare-09-01663-s001.zip › healthcare-1432858-supplementary.pdf]

Table S1. Search command.

Database	Search Command
Pubmed	("alveolar bone"[Title/Abstract] OR "alveolar plate"[Title/Abstract] OR (("alveolar"[All Fields] OR "alveolarization"[All Fields] OR "alveolars"[All Fields]) AND "shelf"[Title/Abstract]) OR "buccal bone"[Title/Abstract] OR "buccal plate"[Title/Abstract] OR "buccal shelf"[Title/Abstract] OR "facial bone"[Title/Abstract] OR "facial plate"[Title/Abstract] OR (("face"[MeSH Terms] OR "face"[All Fields] OR "facial"[All Fields] OR "facials"[All Fields]) AND "shelf"[Title/Abstract]) OR "bundle bone"[Title/Abstract] OR "alveolar crest"[Title/Abstract] OR "alveolar ridge"[Title/Abstract] OR "alveolar bone thickness"[Title/Abstract] OR "alveolar bone width"[Title/Abstract] OR (("alveolar"[All Fields] OR "alveolarization"[All Fields] OR "alveolars"[All Fields]) AND "plate thickness"[Title/Abstract]) OR (("alveolar"[All Fields] OR "alveolarization"[All Fields] OR "alveolars"[All Fields]) AND "plate width"[Title/Abstract]) OR ((("alveolar"[All Fields] OR "alveolarization"[All Fields] OR "alveolars"[All Fields]) AND "shelf"[All Fields]) AND "thickness"[Title/Abstract]) OR (("alveolar"[All Fields] OR "alveolarization"[All Fields] OR "alveolars"[All Fields]) AND "shelf width"[Title/Abstract]) OR "buccal bone thickness"[Title/Abstract] OR "buccal bone width"[Title/Abstract] OR "buccal plate thickness"[Title/Abstract] OR "buccal plate width"[Title/Abstract] OR ((("buccal"[All Fields] OR "buccally"[All Fields]) AND "shelf"[All Fields]) AND "thickness"[Title/Abstract]) OR (("buccal"[All Fields] OR "buccally"[All Fields]) AND "shelf width"[Title/Abstract]) OR "facial bone thickness"[Title/Abstract] OR (("face"[MeSH Terms] OR "face"[All Fields] OR "facial"[All Fields] OR "facials"[All Fields]) AND "bone width"[Title/Abstract]) OR "facial plate thickness"[Title/Abstract] OR (("face"[MeSH Terms] OR "face"[All Fields] OR "facial"[All Fields] OR "facials"[All Fields]) AND "plate width"[Title/Abstract]) OR ((("face"[MeSH Terms] OR "face"[All Fields] OR "facial"[All Fields] OR "facials"[All Fields]) AND "shelf"[All Fields]) AND "thickness"[Title/Abstract]) OR (("face"[MeSH Terms] OR "face"[All Fields] OR "facial"[All Fields] OR "facials"[All Fields]) AND "shelf width"[Title/Abstract]) OR (("bundle"[All Fields] OR "bundle s"[All Fields] OR "bundled"[All Fields] OR "bundles"[All Fields] OR "bundling"[All Fields]) AND "bone thickness"[Title/Abstract]) OR (("bundle"[All Fields] OR "bundle s"[All Fields] OR "bundled"[All Fields] OR "bundles"[All Fields] OR "bundling"[All Fields]) AND "bone width"[Title/Abstract]) OR "alveolar crest thickness"[Title/Abstract] OR "alveolar crest width"[Title/Abstract] OR "alveolar ridge thickness"[Title/Abstract] OR "alveolar ridge width"[Title/Abstract])<br>AND ("Tooth"[Title/Abstract] OR "Teeth"[Title/Abstract] OR "maxilla*"[Title/Abstract] OR "mandib*"[Title/Abstract] OR "Incisor"[Title/Abstract] OR "Canine"[Title/Abstract] OR "Premolar"[Title/Abstract] OR "Molar"[Title/Abstract])<br>AND ("cone beam computed tomography"[MeSH Terms] OR "CT"[Title/Abstract] OR "CBCT"[Title/Abstract] OR "Tomography"[Title/Abstract] OR "cone beam computed tomography"[Title/Abstract] OR "computed tomography"[Title/Abstract] OR "ct imaging"[Title/Abstract] OR "cbct imaging"[Title/Abstract])<br>Filter: "Humans"
Medline	#1 (alveolar bone):ti,ab,kw<br>#2 (alveolar plate):ti,ab,kw<br>#3 (alveolar shelf):ti,ab,kw<br>#4 (buccal bone):ti,ab,kw<br>#5 (buccal plate):ti,ab,kw<br>#6 (buccal shelf):ti,ab,kw<br>#7 (facial bone):ti,ab,kw<br>#8 (facial plate):ti,ab,kw<br>#9 (facial shelf):ti,ab,kw<br>#10 (bundle bone):ti,ab,kw<br>#11 (alveolar crest):ti,ab,kw<br>#12 (alveolar ridge):ti,ab,kw<br>#13 (alveolar bone thickness):ti,ab,kw<br>#14 (alveolar bone width):ti,ab,kw<br>#15 (alveolar plate thickness):ti,ab,kw<br>#16 (alveolar plate width):ti,ab,kw

#17 (alveolar shelf thickness):ti,ab,kw
#18 (alveolar shelf width):ti,ab,kw
#19 (buccal bone thickness):ti,ab,kw
#20 (buccal bone width):ti,ab,kw
#21 (buccal plate thickness):ti,ab,kw
#22 (buccal plate width):ti,ab,kw
#23 (buccal shelf thickness):ti,ab,kw
#24 (buccal shelf width):ti,ab,kw
#25 (facial bone thickness):ti,ab,kw
#26 (facial bone width):ti,ab,kw
#27 (facial plate thickness):ti,ab,kw
#28 (facial plate width):ti,ab,kw
#29 (facial shelf thickness):ti,ab,kw
#30 (facial shelf width):ti,ab,kw
#31 (bundle bone thickness):ti,ab,kw
#32 (bundle bone width):ti,ab,kw
#33 (alveolar crest thickness):ti,ab,kw
#34 (alveolar crest width):ti,ab,kw
#35 (alveolar ridge thickness):ti,ab,kw
#36 (alveolar ridge width):ti,ab,kw
#37 #1 OR #2 OR #3 OR #4 OR #5 OR #6 OR #7 OR #8 OR #9 OR #10 OR #11 OR #12 OR #13 OR #14 OR #15 OR #16 OR #17 OR #18 OR #19 OR #20 OR #21 OR #22 OR #23 OR #24 OR #25 OR #26 OR #27 OR #28 OR #29 OR #30 OR #31 OR #32 OR #33 OR #34 OR #35 OR #36
#38 (Tooth):ti,ab,kw
#39 (teeth):ti,ab,kw
#40 (Maxilla*):ti,ab,kw
#41 (Mandib*):ti,ab,kw
#42 (Incisor):ti,ab,kw
#43 (Canine):ti,ab,kw
#44 (Premolar):ti,ab,kw
#45 (Molar):ti,ab,kw
#46 #38 OR #39 OR #40 OR #41 OR #42 OR #43 OR #44 OR #45
#47 (cone beam computed tomography):ti,ab,kw
#48 (CT):ti,ab,kw
#49 (CBCT):ti,ab,kw
#50 (Tomography):ti,ab,kw
#51 (Computed tomography):ti,ab,kw
#52 (CT imaging):ti,ab,kw
#53 (CBCT imaging):ti,ab,kw
#54 #47 OR #48 OR #49 OR #50 OR #51 OR #52 OR #53
#55 #37 AND #46 AND #54

Table S3. Risk of bias analysis.

Study	Randomization	Blinding	Appropriate and clearly focused question of the Study	Defined criteria for 1. Inclusion 2. Exclusion	Appropriate No. of patients	Conflict of interest (COI) was stated	Source of Funding	Risk of bias
**Adiguzel 2017**	NA	NA	Yes	1. Yes<br>2. Yes	113	No COI claimed	NA	High
**Al-Jandan 2013**	No	NA	Yes	1. Yes<br>2. Yes	50	No COI claimed	No	High
**AlMasri 2015**	Yes	NA	Yes	1. Yes<br>2. No	16	No COI claimed	No	High
**AlTarawneh 2017**	NA	NA	Yes	1. Yes<br>2. Yes	120	No COI claimed	NA	High
**Amid 2017**	NA	NA	Yes	1. Yes<br>2. Yes	144	NA	No	High
**Behnia 2015**	Yes	NA	Yes	1. Yes<br>2. No	18	No COI claimed	NA	High
**Botelho 2020**	NA	NA	Yes	1. Yes<br>2. Yes	87	No COI claimed	NA	High
**Chen 2017**	NA	NA	Yes	1. Yes<br>2. Yes	16	No COI claimed	Funded	High
**D'Silva 2019**	NA	NA	Yes	1. Yes<br>2. Yes	66	No COI claimed	NA	High
**Demircan 2015**	NA	NA	Yes	1. Yes<br>2. Yes	60	NA	NA	High
**El Nahass 2013**	NA	NA	Yes	1. Yes<br>2. Yes	93	NA	NA	High
**Eraydin 2017**	Yes	NA	Yes	1. Yes<br>2. Yes	24	No COI claimed	No	Moderate
**Farahamnd 2017**	Yes	NA	Yes	1. Yes<br>2. Yes	132	NA	NA	Moderate
**Foosiri 2018**	Yes	NA	Yes	1. Yes<br>2. Yes	51	No COI claimed	No	Moderate
**Gakonyo 2018**	NA	NA	Yes	1. Yes<br>2. Yes	184	No COI claimed	NA	High
**Ganji 2017**	No	NA	Yes	1. Yes<br>2. Yes	32	No COI claimed	No	High
**Gluckman 2017**	NA	Yes	Yes	1. Yes<br>2. Yes	150	NA	NA	High
**Januário 2011**	NA	NA	Yes	1. Yes<br>2. No	250	NA	NA	High
**Jin 2005**	No	NA	Yes	1. Yes<br>2. Yes	66	NA	NA	High
**Kheur 2015**	No	Yes	Yes	1. Yes	150	No COI claimed	No	Moderate

				2. Yes				
**Khoury 2016**	NA	NA	Yes	1. No<br>2. Yes	47	No financial interest claimed	NA	High
**Lau 2011**	Yes	NA	Yes	1. Yes<br>2. Yes	170	NA	NA	High
**Lee 2019**	NA	NA	Yes	1. Yes<br>2. Yes	20	No COI claimed	No	High
**Lin 2018**	NA	NA	Yes	1. Yes<br>2. Yes	21	No COI claimed	Funded	High
**López-Jarana 2018**	NA	Yes	Yes	1. Yes<br>2. Yes	49	No COI claimed	Funded	Moderate
**Matsuda 2016**	NA	Yes	Yes	1. Yes<br>2. Yes	95	No financial interest claimed	NA	Moderate
**Nahás-Scocate 2014**	No	NA	Yes	1. Yes<br>2. Yes	30	NA	NA	High
**Nowzari 2010**	Yes	NA	Yes	1. Yes<br>2. Yes	101	NA	Funded	Moderate
**Nucera 2017**	Yes	Yes	Yes	1. Yes<br>2. Yes	30	NA	NA	Low
**Park 2014**	NA	NA	Yes	1. Yes<br>2. Yes	20	No COI claimed	NA	High
**Pascual 2017**	NA	NA	Yes	1. No<br>2. Yes	15	No COI claimed	NA	High
**Porto 2019**	NA	NA	Yes	1. Yes<br>2. Yes	422	No COI claimed	Funded	High
**Ramanauskaite 2020**	NA	NA	Yes	1. Yes<br>2. No	60	No COI claimed	Funded	High
**Rojo-Sanchis 2017**	NA	NA	Yes	1. Yes<br>2. Yes	44	No COI claimed	NA	High
**Sendyk 2015**	NA	NA	Yes	1. Yes<br>2. No	35	No COI claimed	NA	High
**Shrestha 2019**	Yes	NA	Yes	1. Yes<br>2. Yes	146	No COI claimed	Funded	Moderate
**Temple 2015**	NA	NA	Yes	1. Yes<br>2. Yes	265	No COI claimed	NA	High
**Üner 2019**	NA	NA	Yes	1. No<br>2. Yes	160	No COI claimed	No	High
**Wang 2014**	NA	NA	Yes	1. Yes<br>2. No	300	No COI claimed	Funded	High
**Yoshimine 2012**	NA	NA	Yes	1. No<br>2. Yes	30	No COI claimed	No	High
**Younes 2015**	Yes	NA	Yes	1. Yes<br>2. Yes	21	COI claimed: collaboration agreements	Funded	High

**Yuan 2018**	NA	NA	Yes	1. No<br>2. No	40	NA	NA	High
**Zahedi 2018**	NA	NA	Yes	1. No<br>2. Yes	170	No COI claimed	NA	High
**Zekry 2012**	Yes	NA	Yes	1. Yes<br>2. Yes	200	No COI claimed	NA	Moderate
**Zhang 2015**	Yes	NA	Yes	1. Yes<br>2. Yes	105	No COI claimed	Funded	Moderate
